# microRNAs Biogenesis, Functions and Role in Tumor Angiogenesis

**DOI:** 10.3389/fonc.2020.581007

**Published:** 2020-11-27

**Authors:** Tiziana Annese, Roberto Tamma, Michelina De Giorgis, Domenico Ribatti

**Affiliations:** Department of Basic Medical Sciences, Neurosciences and Sensory Organs, Section of Human Anatomy and Histology, University of Bari Medical School, Bari, Italy

**Keywords:** angiogenesis, lymphoma, microenvironment, microRNA, tumor progression

## Abstract

microRNAs (miRNAs) are small non-coding RNA molecules, evolutionary conserved. They target more than one mRNAs, thus influencing multiple molecular pathways, but also mRNAs may bind to a variety of miRNAs, either simultaneously or in a context-dependent manner. miRNAs biogenesis, including miRNA transcription, processing by Drosha and Dicer, transportation, RISC biding, and miRNA decay, are finely controlled in space and time.

miRNAs are critical regulators in various biological processes, such as differentiation, proliferation, apoptosis, and development in both health and disease. Their dysregulation is involved in tumor initiation and progression. In tumors, they can act as onco-miRNAs or oncosuppressor-miRNA participating in distinct cellular pathways, and the same miRNA can perform both activities depending on the context.

In tumor progression, the angiogenic switch is fundamental. miRNAs derived from tumor cells, endothelial cells, and cells of the surrounding microenvironment regulate tumor angiogenesis, acting as pro-angiomiR or anti-angiomiR.

In this review, we described miRNA biogenesis and function, and we update the non-classical aspects of them. The most recent role in the nucleus, as transcriptional gene regulators and the different mechanisms by which they could be dysregulated, in tumor initiation and progression, are treated. In particular, we describe the role of miRNAs in sprouting angiogenesis, vessel co-option, and vasculogenic mimicry. The role of miRNAs in lymphoma angiogenesis is also discussed despite the scarcity of data.

The information presented in this review reveals the need to do much more to discover the complete miRNA network regulating angiogenesis, not only using high-throughput computational analysis approaches but also morphological ones.

## Introduction

From the discovery of the lin-4/lin-14 paradigm in Caenorhabditis elegans, microRNAs (miRNAs) have come a long way (1–4). miRNAs were found in eukaryotic organisms, viruses, and prokaryotic cells.

miRNAs of viral origin, known as v-miRNAs, in the host, can function as post-transcriptional gene regulators as well as viral genes. Their expression can prolong the longevity of infected cells, enhance their immune response evasion, and regulate the switch to lytic infection (1–4). miRNAs in prokaryotic cells is a more recent and, therefore, unexplored research field. They are comparable in size and can be export outside the cells by vesicles as eukaryotic miRNAs, but diverge considerably from these in biogenesis and presentation to the target mRNAs (5).

miRNAs are non-protein-coding regulatory genes, evolutionary conserved, partially complementary to one or more messenger RNA (mRNA) molecules (target mRNAs) (6). Mature miRNAs are short, single-stranded RNA molecules of about 21-23 nucleotides in length, which is just about right for specificity to particular target genes as well as for plasticity to evolve towards new gene targets. They regulate the expression of homologous target-gene transcripts, at transcriptional and/or post-transcriptional level, through a mechanism known as RNA interference (RNAi). They may induce both down- and up-gene expression by different mechanisms in both cytoplasm and nucleus where they have been found.

In this review, we described miRNA biogenesis, regulation, and function underline essential aspects of processes localization and timeline. Indeed, we update the non-classical aspects of miRNAs, such as their new role in the nucleus as transcriptional gene regulators. Then different mechanisms by which miRNAs could be dysregulated in tumor initiation and progression are addressed. In particular, we describe the role of miRNA in sprouting angiogenesis, vessel co-option, and vasculogenic mimicry. The role of miRNA in lymphoma angiogenesis is also discussed despite the scarcity of data.

In the end, this review demonstrates the need to do much more to discover the complete miRNA network regulating angiogenesis, not only using high-throughput analysis approaches but also morphological ones.

### miRNA Biogenesis

miRNAs biogenesis starts from DNA sequences, called miRNA genes, or from clusters genes which are only transcripted as miRNA molecules or together as polycistronic transcripts, respectively (7, 8). Alternatively, the miRNAs localize within an intron or untranslated region (UTR) of a protein-coding gene (8). Two main pathways of biogenesis were identified as canonical and non-canonical ones.

### Canonical miRNA Biogenesis and Functions

In the canonical biogenesis pathway, primary miRNAs (pri-miRNA) are transcribed from their genes by RNA polymerase III, and then processed into precursor miRNA (pre-miRNAs) by the Microprocessor complex, consisting of two multiprotein units (9–11). One of these is a large multiprotein unit that includes different classes of RNA-associated proteins, double-stranded RNA (dsRNA) binding proteins, ribonucleoproteins, and Ewing’s sarcoma protein family. The other one is a small multiprotein unit that includes the RNase III enzyme, named Drosha, and the RNA binding protein DiGeorge Syndrome Critical Region 8 (DGCR8) (12).

The Microprocessor recognizes specific motifs within the pri-miRNA, and cleaves it at the base of the characteristic hairpin structure, generating a 5′-monophosphate and a 3′-2-nt overhang on pre-miRNA (10, 13, 14). After, pre-miRNAs are exported, from the nucleus to the cytoplasm, by a complex of exportin5 (XPO5) and RAS-related nuclear protein-guanosine-5’-triphosphate-ase (Ran-GTPase) (11). In the cytoplasm, the terminal loop of pre-miRNA is removed by the RNase III endonuclease Dicer (13).

At this point, miRNAs appear as short RNA duplexes termed miRNA duplexes that are loaded into the RNA-induced silencing complex (RISC) (15). This machine is a multiprotein complex that includes proteins such as Argonaute RISC Catalytic Component 2 (Ago2), Aubergine (another Ago family protein) Staphylococcal Nuclease Domain-Containing Protein 1 (SND1), Astrocyte Elevated Gene-1 (AEG-1), Fragile X Mental Retardation 1 (FMR1), VIG (vasa intronic gene), R2D2 (a dsRNA binding protein with two dsRNA binding domains), and Armitage-RNA helicase1 (15–17).

Ago2 is the RISC catalytic component that leaves and/or removes one strand of the duplex (18, 19). Based on the directionality of the miRNA’s strands originated, the name of the mature miRNA will be 5p strand (miRNA-5p) if it arises from the 5′-end of the pre-miRNA hairpin, or will be 3p strand (miRNA-3p) if it originates from the 3′-end. The two mature miRNAs strands have different target-specificity (20, 21). The strand which will remain incorporated into the RISC is the active strand, and it is named the guide strand, leading strand or miR. The other strand gets degraded, and it is named the passenger strand or miR* (22). Thermodynamic features that, in turn, are related to ribonucleotide (purines vs. pyrimidines) composition of the duplex, play an essential role in miRNA-RISC assembly (23).

RISC with the loaded miRNA (miRISC) acts inhibiting multiple steps of protein synthesis and affecting mRNA target stability (24–26). In detail, the miRNA in the RISC complex drives it to an mRNA target in a complementary-nucleotides-based way. Usually, the mRNA target-site is into 3'untranslated region (3’UTR), and the complementary sequence on miRNA, called seed sequence, is located at 2-7 nucleotides. However, non-canonical seed regions have been identified, such as in the 5’UTR sequence and the coding regions of mRNAs (27).

After recognition, target mRNA is still stable, but miRISC prevents its translation by inhibition of translation elongation, or through protein degradation or ribosome drop-off. In the end, mRNA, after deadenylation and decay, is degraded (28, 29). More precisely, to date, it seems that translational repression and target mRNA degradation are spatiotemporal uncoupling events. Concerning the timing, in Drosophila, Zebrafish, and mouse embryonic stem cells (ESCs), it was demonstrated that translational repression, at an early step, always occurred before mRNA degradation (30–33). This result suggests that the block of protein translation is the first event of miRNA-mediated gene silencing (the cause), and the mRNA target degradation is a secondary consequence (the effect). Concerning space, it was demonstrated that RISC assembly, its interaction with miRNA and targeted mRNA, and translational repression are processes compartmentalized on rough endoplasmic reticulum (rER) membrane. On the contrary, the degradation of the mRNAs (deadenylation and decay) takes place on endosome/multivesicular bodies (MVBs) (34, 35).

In addition to the well-known miRNAs cytoplasmic localization and functions, the nuclear ones are noteworthy. Indeed, it has been proven that minimal miRISC complex is also present in mammalian cell nuclei, where it is 20-fold smaller (36, 37). It consists only of Ago2 and miRNA, is loaded in the cytoplasm, and is imported into the nucleus by binding to importin 8 (IPO8) (38). The combination of minimal miRISC complex with Trinucleotide Repeat Containing Adaptor 6A (TNRC6A), which possesses a nuclear localization signal (NLS) and a nuclear export signal (NES), ensure the nuclear-cytoplasmic shuttling (38) even though a more selective mechanism for miRNAs distribution could be operated by nucleotides-motif at the 3’ end of the miRNA.

In the nucleus, miRNAs contribute to the regulation of both coding and non-coding RNA transcriptome (28, 37). They can act in blocking or promoting pri-miRNA maturation regulating long non-coding RNAs (lncRNAs) levels (39–41).

Other nuclear miRNAs colocalize with ribosomal RNAs (rRNAs) in the nucleolus, where they are stored, or in the cytoplasm where they influence the abundance of rRNAs and/or regulate ribosomes interaction with accessory proteins (42–45).

Moreover, in the nucleus, miRNAs induce remodeling of chromatin structure, regulate alternative splicing, and in turn, regulate itself (46–48). miRNAs can also mediate transcriptional gene activation (TGA) or transcriptional gene silencing (TGS) (49, 50). TGA depends on the complementarity between DNA sequences of gene promoter and the 5’-seed with a part of the flanking region of miRNA. Two mechanisms that unlock gene silencing have been identified: i) in case of silencing complexes on gene promoter, such as lncRNA or promoter-associated RNA (pRNA), miRNA can act inducing the cleavage of these molecules by the recruiting of the nuclear RISC; ii) miRNA can directly recognize the nascent RNA promoter and recruit the minimal miRISC that in turn enroll the protein complex assigned to shift the chromatin structure toward a more permissive arrangement (51, 52).

About TGS, where epigenetic modiﬁcations have a more prominent role than in TGA: i) miRNA with its seed sequence may directly interact with single-stranded DNA complementary sequences in the promoter region inducing histone epigenetic modification and altering the binding of transcription factors; ii) miRISC may directly target to non-coding transcripts, sense and antisense, and serves as a molecular scaffold to recruit an inhibitory protein complex and chromatin modulators that will establish a non-permissive transcriptional status; iii) miRNA may hybridize with double-stranded DNA to form relatively stable RNA*DNA : DNA triplexes *via* Hoogsteen or reverse Hoogsteen interaction that induces promoter-speciﬁc transcriptional repression through the disruption of the formation of the pre-initiation complex at the promoter (28, 37).

miRNAs levels and their activity can be regulated by a series of post-translational modifications (PTMs) affecting the miRNA processing machine (see **Table 1** and below section PARylation post-translational modification affect miRNA activity in tumors). Moreover, co- and post-transcriptional regulation of miRNA transcripts are performed by specific RNA-biding proteins (RBPs), which affect miRNA processing and loading into RISC, and facilitate the crosstalk between various RNA pathways [reviewed in (28)].

**Table 1:** Post-translational modifications (PTMs) of miRNA transcripts.

PTMs	Regulated factor			
	*Drosha*	*DGCR8*	*AGO2*	*TRBP*
***Phosphorylation***	In stress conditions, p38 phosphorylates Drosha, reducing Drosha-DGCR8 interaction, and consequently, miRNA levels decreased (53). On the contrary, if p38 activates MAPKAPK2, this, in turn, phosphorylates and activates p68 (an auxiliary component of the Microprocessor complex), inducing the processing of selected pri-miRNA (54). In the case of Drosha phosphorylation at Ser300 or Ser302 by GSK3β, its nuclear localization and increased activity are enhanced (55)	If MAPK hyperphosphorylates DGCR8, miRNA levels increase sustained by a higher Microprocessor complex activity (56). In the case of DNA damage, DGCR8 could be phosphorylated by protein kinase ABL to stimulates pri-miRNA processing (57).	In the hypoxia condition, EGFR phosphorylates AGO2 at Tyr393, reducing its ability to form the loading RISC complex. The effect is a reduced level of selected subsets of miRNA (57).	If ERK phosphorylates TRBP, the complex Dicer-TRPB will be more stable e will favor a pro-growth miRNA expression signature (58). This mechanism can be further supported by the activation of S6K by the same ERK pathway (59).
***Ubiquitination***	mTOR increases the levels of MDM2, which functions as an E3 ubiquitin ligase of Drosha, leading to Drosha proteasome-mediated degradation and thus reduced miRNA processing (60).			
***Sumoylation***		DGCR8 could be sumoylated at Lys, which prevents its ubiquitylation and degradation (61).		TRBP could be sumoylated at Lys52. This PTMs regulates miRNA/siRNA efficiency favoring the Ago2 organization to form the effective RISC for RNAi (62).

Alterations in miRNA biogenesis can promote and support the onset of cancer. The oncogene p53, whose mutations occur in 50% of human cancers, promotes cancer interfering with Drosha in the Microprocessor complex. It inhibits miRNA processing, resulting in the accumulation of pri-miRNAs and parallel depletion of mature miRNAs (63). Mutant p53, by sequestrating the Drosha cofactors, DEAD-box RNA helicases p68 (DDX5), or p72/p82 (DDX17), promotes tumor cell migration, epithelial to mesenchymal transition (EMT), and cell survival (64, 65).

Moreover, cancer is supported by altered expression levels of miRNA processing machinery components such as Drosha, DGCR8, and Dicer that are down-regulated or up-regulated in several cancers (66). For instance, in neuroblastoma and ovarian cancer, Drosha and Dicer’s reduced expression levels and overexpression of DGCR8 correlate with tumor progression and a worse clinical outcome (67–69).

Dicer mutations lead to altered expression levels or could affect the region encoding RNase III domains that cause defects in its function. In several tumors, such as pleuropulmonary blastoma, cystic nephroma, ovarian cancer, Wilms tumor, pituitary blastoma, and rhabdomyosarcoma, the pathogenesis is supported by reduced Dicer expression and/or impaired function that in turn induce an aberrant miRNA expression (70–73).

Impaired function of the Dicer cofactor TRBP also contributes to miRNA dysregulation in cancer. In the Ewing sarcoma family tumor, it was demonstrated that cancer stem cell renewal and tumor maintenance depend on TRBP expression (74).

Pre-miRNA export dysfunction may lead to tumorigenesis. By cancer tissue microarrays (TMAs) and the cancer genome atlas (TCGA) RNA seq-data, it was shown that increased level of XPO5 promotes and sustain tumor including prostate cancer, breast cancer, ovarian cancer, and bladder cancer (75–77).

### Non-Canonical miRNA Biogenesis and Functions

In addition to the canonical pathway, miRNAs could be processed from unexpected non-coding RNAs by mechanisms that may depend on cell type and cellular state (78).

In the non-canonical miRNA biogenesis pathway, functionally miRNAs result from a different combination of the same proteins of the canonical pathway. Two main non-canonical miRNA biogenesis pathways are distinguished: Drosha/DGCR8-independent and Dicer-independent (79). An example of the first is miRtrons biogenesis (80). MiRtron-derived pri-miRNAs correspond to the entire intronic sequence of the mRNA encoding genes in which they are located. They are processed by splicing and not by the Microprocessor complex (81). After splicing, miRtron-derived pre-miRNAs are exported to the cytoplasm by XPO5 and are processed by Dicer to form a mature miRNA (82, 83). miRtrons could be distinguished from canonical miRNAs looking for key features such as bulges in the stem region, guanine content, hairpin free energy, and hairpin length (84).

An example of a non-canonical miRNA biogenesis Dicer-independent pathway is pre-miR-451. It is processed, initially, by Drosha to liberate the pre-miRNA, following, in the cytoplasm, it is loaded into Ago2 to catalyze the maturation of this microRNA (85). Pre-miR-451 is not processed by Dicer because its stem-loop structure is too short to be efficiently recognized and processed (86, 87). Therefore, in this mechanism, it seems that Ago2 is sufficient for RISC loading and proper guide strand selection (88). This miRNA is involved in tumorigenesis and tumor progression of several cancers. It is downregulated in epithelial cells cancer that lose their basolateral polarity, dramatically proliferate, and turn to invasive adenocarcinoma forms as demonstrated in gastric cancer, colorectal cancer, non–small cell lung carcinoma (NSCLC) (89–91)

Non-canonical miRNA could also be small nucleolar RNAs (snoRNAs) and transfer RNAs (tRNAs) (78). snoRNA-derived miRNAs are approximately 22 nucleotides in length, require Dicer activity, but are independent of Drosha/DGCR8, bind to Argonaute proteins and repress target mRNAs as miRNA do (92, 93).

tRNAs with their clover shape can generate many non-coding small RNAs, named tRNA-derived small RNAs (tsRNAs). These, based on their mechanism of biogenesis and length, are classified as tRNA-derived stress-induced RNA (tiRNA) or tRNA-derived fragment (tRF). tRFs are smaller than tiRNAs, originate from a specific sequence of mature or primary tRNAs cleaved by Dicer, or they could be processed by RNase Z or ELAC2 in the nucleus and cytoplasm, respectively (94). They can bind to the Ago proteins family in a cell-type-specific manner and can regulate mRNA stability by directly binding to it, inhibiting protein translation, or cleaving a partially complementary target site (94–96).

The role of non-canonical miRNA in the biological functions and development of cancer is poorly understood. Splicing factors, such as SRSF1 or SRSF2, alter the levels of miRNAs of miRtron origin. In the colorectal cancer cell line HCT116, it was found that increased nuclear levels of SRSF1 positively correlate to hsa-miR-1229-3p expression, while increased nuclear levels of SRSF2 positively correlate to hsa-miR-1227-3p and hsa-miR-1229-3p expression (97). The miRtron miR-6778–5p, derived from intron 5 of SHMT1 (coding cytoplasmic serine hydroxymethyltransferase), is a pivotal regulator of cancer stem cell stemness in Drosha-silenced gastric cancer cells. It promotes SHMT1 expression that sustains cancer energy intake *via* folate-dependent serine/glycine inter-conversion in the one-carbon mitochondrial metabolic pathway (98).

By RNA-seq-based data set, in MDA-MB-231s breast cancer cell line and more than 90% of Luminal B Her2^+^ human breast cancer, a small nucleolar RNA-derived RNAs, snoRNA-93, was identified as a promoter of invasion (99). In prostate cancer, the increased expression of small nucleolar RNA-derived RNAs snoRD78 was detected in a subset of patients that developed the metastatic disease (100).

## PARylation Post-Translational Modification Affect miRNA Activity in Tumors

Among the PTMs affecting the miRNA processing machine, poly-ADP-ribosylation (PARylation) is critical. It is a mechanism by which poly-ADP-ribose (pADPr) macromolecular polymer is added to some proteins, acting as a post-translational modification well documented in the nucleus and the cytoplasm (101–103). The reactions are catalyzed by ADP-ribosyltransferases proteins that include poly-ADP-ribose polymerases (PARPs) (104). PARPs are involved in DNA repair, when DNA single-strand breaks are present, and induce apoptosis *via* exhaustion of ATP reserves (105, 106). To date, knowledge on the role of PARPs in RNA metabolism is growing (107–109). In 2011, Leung et al. demonstrated that pADPr is a crucial regulator of miRNAs PTMs in the cytoplasm and, consequently, mRNA expression levels (110). In detail, by immunoprecipitation assay and GFP fusion technology under different experimental conditions in four human cell lines, they showed that cytoplasmic stress granules were rich in mRNA binding proteins, contained six poly-ADP-ribose polymerases, two poly-ADP-ribose glycohydrolase, and Ago proteins (110, 111). Ago2, in standard and stress conditions, were PARylated by catalytically inactive PARP13 and other synthesizing PARPs (mono and poly-ADPr), but during stress, it was much more PARylated, probably for increased PARP activity and/or decreased poly-ADP-ribose glycohydrolase (PARG) activity. Ago2 increased PARylation reduces the miRNA repression activity and miRNA-directed cleavage due to disruption of electrostatic interaction between miRNA:mRNA or steric obstacles for effective silencing of miRNAs (110).

In the colorectal cancer DLD-1 cell line was found that PARylation of Ago2-associated proteins during viral infection relieves miR-17/93 family repression of the interferon-stimulated genes which contain in the 3′ UTRs the miRNA target sites. This means that cells respond to viral infection by downregulation of miRNAs pathway activity *via* the PARylation of Ago2 complexes (112, 113). By immunohistochemistry reactions on TMAs containing tumor and normal tissue, reduced expression of PARP13 was demonstrated in liver, colon, and bladder cancers (114). PARP13 targeted TNF-related apoptosis-inducing ligand (TRAIL) 4 transcript at the cell decay pathway, destabilizing its mRNA after transcription, *via* exosome, by binding to its 3 ‘UTR region, and thus increasing the sensitivity of tumor cells to TRAIL-mediated apoptosis (115). A PARP13 anticancer role was also found in Hepatitis B Virus related-liver cancer and leukemia, thanks to its antiviral activity (116, 117). PARP1 expression was found to increase in breast, uterine, lung cancer, ovarian cancer, skin cancer, and non-Hodgkin’s lymphoma (118–120). In breast cancers, including triple-negative breast cancers, PARP1 expression negatively correlates to the estrogen receptor, progesterone receptor, or epidermal growth factor receptor-2 expression (121). In breast cancer, the oncomir miR-155-5p was found mostly upregulated and associated with a more aggressive type and worse outcome (122). The combined treatment with Olaparib, an inhibitor of PARP1, with miR-155-5p ectopic overexpression was found a great combination to induce lethal effect to reduce cancer cells viability (123)

Further investigation of PARPs as a biomarker for novel cancer therapies is needed.

### miRNA in Tumors

Tumors develop due to progressive DNA alterations, which will allow tumor cells to proliferate uncontrollably (124). Tumor cells acquire the ability not to respond to growth inhibitors and to resist to mechanisms of programmed cell death. Therefore, tumor cells become immortal and able to shift their metabolism to more advantageous energy mechanisms in the interest of continuous growth and proliferation. Furthermore, to grow beyond a specific size, tumors activate angiogenesis mechanisms, which will allow the tumor cells to receive more oxygen and nutrients, and to eliminate metabolism waste more efficiently. Thanks to angiogenesis, tumor cells not only proliferate but becomes able to invade the surrounding tissues giving rise to local metastases. Moreover, tumor initiation and progression are supported and favored by the inflammation processes, and by the tumor cell competence to escape the immune surveillance.

In this complex genetic disease, coding and non-coding genes are involved. miRNAs, because of their ubiquitous role in gene regulation, are critical regulators in various biological processes, such as differentiation, proliferation, apoptosis, and development in both health and disease (125).

The activity of miRNAs in tumors is regulated by the same alterations affecting protein-coding genes, such as chromosomal rearrangements, genomic amplifications or deletions or mutations, abnormal transcriptional control, dysregulation of epigenetic changes and defects in the biogenesis machinery. A typical chromosomal rearrangement is a chromosomal translocation, especially in hematological malignancies, in which it promotes tumor development and progression by the promoter exchange or by the creation of chimeric genes translated as fusion proteins. In Acute Myeloid Leukemia (AML) patients with myeloid/lymphoid leukemia gene (or mixed-lineage leukemia, MLL) rearrangement, by large-scale genome-wide microarray analysis, it was demonstrated that among 48 selected miRNAs, 47 of them are increased (126). This result suggests that MLL fusion protein promotes the transcription of its downstream targets, such as miRNA genes.

The primary miRNAs alteration in tumors is the aberrant gene expression arises from amplification or deletion of specific genomic regions that coupling with abnormal expression levels of mature and/or precursor miRNA compared with the corresponding healthy tissues. In B-cell chronic lymphocytic leukemia (B-CLL), the miR-15a/16-1 cluster gene is deleted in more than 50% of cases, resulting in decreased expression of both miRNAs (127). Single-nucleotide polymorphisms (SNPs) in miRNAs or miRNAs-targets are associated with the development of several tumors. For example, in breast cancer, polymorphisms in the 3′-UTR of the ErbB4 gene could affect miRNA binding sites resulting in post-translational dysregulation of mRNA and a predisposition to tumor development (128). In gastric cancer, SNPs in miRNA machinery genes was strictly associated with cancer susceptibility and malignant behavior (*Dicer* and *GEMIN4*), or lymphatic metastasis (*GEMIN4* and *Ago1*) (129). In hepatocellular carcinoma, SNPs in miRNA3152 and miRNA449b were linked with the risk of tumor development (130).

Different transcription factors, such as p53, c-Myc, nuclear receptors, and RAS control miRNA expression and are often associated with tumorigenesis. For instance, p53 strengthens his tumor suppressor activity, also inducing the expression of specific miRNAs, which show the same suppressive functions. In pancreatic cancer cells, p53 directly transactivates miR34a inducing gene expression reprogramming and promoting apoptosis (131). On the contrary, there are oncogenic transcription factors that induce tumorigenesis, such as members of the RAS family. miR-143/miR-145 co-expressed in the same primary transcript, highly expressed in healthy colon, are significantly decreased in colorectal cancer in which are targeted by KRAS (132).

Dysregulated epigenetic changes of miRNA may induce tumors. The activation of pri-miRNA transcription is under epigenetic control, especially, it is ruled by promoter-associated CpG island methylation and histone modification. miR34a and other members of the same family, despite being located on different chromosomes, were found hypermethylated in both solid and hematological tumors (133, 134). The use of epigenetic drugs also demonstrates the epigenetic control of miRNA. For example, in triple-negative breast cancer cell lines it was shown that the epigenetic therapy with suberoylanilide hydroxamic acid (SAHA), a histone deacetylase (HDAC) inhibitor, in combination with epigallocatechin-3-gallate (EGCG), a DNA methyltransferase (DNMT) inhibitor, reduces the expression of oncogenic miRNA-221/222 (135).

Dysregulation of all miRNA biogenesis steps may contribute to tumor growth and spread (136). About the Microprocessor complex, both Drosha up- and down-regulation were found in different tumors such as advanced cervical squamous cell carcinoma and clear renal cell carcinoma (137, 138). Further, pre-miRNA export, Dicer, and Ago2 dysregulation are involved. Mutation of XPO5 causes the defect of pre-miRNA export, leading to its accumulation in the nucleus (139). *In vitro* and in an animal model of thyroid cancer, it was shown that oncogenic miR-146b-5p down-regulates miRNA biosynthesis by targeting Dicer and reducing its expression (140). Dicer deletion and/or down-expression promote tumor cell growth, migration, invasion, and EMT. The master regulator of miRNA maturation and function Ago2 was overexpressed in different tumors in which miRNAs act repressing their targets (141–143).

Additionally, nuclear miRNAs in tumor cells is documented. They are involved in the transcriptional regulation of a variety of tumor promoter/repressor genes or tumor-related genes that control tumor initiation, self-sustenance, apoptosis, metastasis, and angiogenesis (37). For example, miR-215-5p, which is nuclear-localized, is up-regulated in high-grade gliomas and promotes tumor cell proliferation, clone formation, migration, and suppresses apoptosis by directly binding to both the promoter and 3’UTR of PCDH9 gene (a member of the protocadherin family and cadherin superfamily). Therefore, PCDH9 expression will be down-regulated at the transcriptional and post-transcriptional levels in glioma (144). Other nuclear-localized miRNAs that promote transcriptional activation of oncogenes by binding to their promoters include miR-483 that binds to IGF2 promoter to increase its expression in Wilms’ tumors and miR-558 that binds to heparanase promoter to enhance its expression in neuroblastoma cells (145, 146). In silico and after mouse model validation, it was demonstrated that miR-744 promotes the transcription of Cyclin B1, favoring the recruitment of RNA polymerase II and the trimethylation of histone-3 at lysine-4 at the promoter region. If it is expressed for a short time, this miRNA promotes cell proliferation. If expressed for a long time, it causes aberrant chromosome distribution and *in vivo* tumor suppression (147). miR-10a acts as a transcription repressor of Hoxd4 in the nucleus of HCT116 colon cancer, THP-1 acute monocytic leukemia, MCF7, and MDA-MB-23 breast cancer cel1 lines. miR-10a induces hypermethylation and trimethylation of histone-3 in the Hoxd4 promoter, thus preventing cancer cell invasion and metastasis (148–150). miR-370, miR-1180, and miR-1236 expression levels are decreased in bladder cancer tissues. These nuclear miRNAs activate tumor suppressor gene p21 by binding to its promoter, as demonstrated in T24 and EJ bladder cancer cell lines (151). In chronic lymphocytic leukemia, the nuclear miRNA-709 controls the biogenesis of tumor suppressor miRNA miR-15a/16-1 (152). It directly binds to a 19-nt recognition element on pri-miR-15a/16-1 and prevents its processing into pre- miR-15a/16-1 and leads to maturation suppression. During apoptotic stimuli, the nuclear level of miRNA-709 decreased because it is exported in the cytoplasm while the nuclear level of miR-15a/16-1 increased, leading to enhanced cell apoptosis *via* anti-apoptotic gene Bcl-2 downregulation (40). The nuclear miR-223, a regulator of innate immunity, is associated with carcinogenesis. In AML, its down-expression induces an increased level of transcriptional factor E2F1, which further reduces miR-223 by binding to its promoter (153). However, the nuclear-localized miRNAs mechanisms of action are incompletely understood and have so far been largely neglected. Further investigations will better elucidate the mechanisms through which nuclear miRNAs execute their functions in different cell types and physiological/pathological conditions.

The pivotal role of miRNAs in tumors is not just the intracellular one (cytoplasm and nucleus). They were also found in extracellular fluids, as free circulating molecules or enclosed in exosomes. These extracellular molecules are intimately involved in cell-cell communication, can travel long distances to affect recipient cells, in particular, immune cells in the tumor microenvironment (154–156). Quality and quantity dysregulations of circulating miRNAs are associated with tumor origin, invasion, metastasis, angiogenesis, immune escape, metabolic switch, and chemosensitivity or chemoresistance (157, 158). Circulating free miRNAs can be released from all cell types as complexes associated with Argonaute proteins during cellular processes such as secretion, apoptosis, inflammation, and necrosis. For instance, by microarrays, in malignant tissue and plasma of tongue squamous cell carcinoma patients, it was shown that many miRNAs (miR-19a, miR-27b, miR-20a, miR-28-3p, miR-200c, miR-151-3p, miR-223, and miR-20b) were up-regulated in plasma, free and exosomes in comparison to benign tongue tissue and plasma (159).

Moreover, in breast cancer *in vitro* model, it was shown that secreted miRNAs from metastatic cells, in a process dependent on Neutral Sphingomyelinase 2 (nSMase2), were transported to endothelial cells by exosomes to promote angiogenesis (160).

In hematological malignancies, tumor-derived exosomes reprogram bone marrow stromal cells, promoting a tumor-supportive microenvironment by suppressing anti-tumor immunity and promoting inflammatory cell recruitment, angiogenesis, osteoclast differentiation, and drug resistance. Moreover, we have demonstrated that in multiple myeloma, cell-derived exosomes induce overexpression of miR-27b-3p and miR-214-3p in fibroblasts of the surrounding tumor microenvironment. These miRNAs increase proliferation and reduce apoptosis of fibroblasts *via* FBXW7 and PTEN signaling, and sustain tumor growth and progression by drug resistance (161, 162).

Summing up, miRNAs in tumors can act as oncogenes or tumor suppressors (163). However, the same miRNA can act as onco-miRNAs and oncosuppressor-miRNAs participating in distinct pathways that have different effects on cell survival, growth, and proliferation depending on the cell type and the pattern of gene expression (164). Another critical aspect of evaluating is the different expression between precursor miRNA and its active molecule. In various types of tumors, it was found altered levels of pre-miRNA but not of the active molecules (165, 166).

So ultimately, all tumors present speciﬁc signatures of miRNAs altered expression. For this reason, the tumor-specific miRNAs expression proﬁle may represent a valid and useful biomarker for diagnosis, prognosis, follow-up, patient stratiﬁcation, deﬁnition of risk groups, and for the development of new therapeutic strategy (167). However, there are many challenges to be addressed before reaching full approval for clinical use (168). It is imperative to validate targets, prevent unwanted off-target effects, and develop an efﬁcient and speciﬁc miRNA delivery system.

### miRNA in Angiogenesis—The AngiomiR

Angiogenesis is the process by which new blood vessels originate. It is a physiological process during embryonic development and reproduction (corpus luteum formation), but it has a pivotal role also in pathological processes such as wound healing, inflammation, and tumor. In this last, angiogenesis is a key process for development and progression, leading to form an eﬃcient vasculature network that guarantees a high supply of nutrients, an efficient system to remove catabolites, and the cancer stem cell maintenance (169). Different mechanisms can contribute to the formation of the new vascular network in tumors, such as sprouting angiogenesis, intussusceptive angiogenesis, vascular co-option, vasculogenic mimicry, postnatal vasculogenesis, or by diﬀerentiation of putative cancer stem cells (CSCs) into endothelial cells (ECs) (170–172).

Angiogenesis is a multi-step process. Taking as an example the well-known sprouting angiogenesis, it begins with activation of ECs, which become able to exit their vessel of origin, invade the surrounding stroma by local enzymatic degradation of the basement membrane, proliferate and migrate in the direction of the angiogenic stimulus, and form tubes. Then the processes of blood vessel fusion, blood vessel pruning, and pericytes stabilization occur to form and complete the new capillary network.

Tumors are heterogeneous and entropic organs, in which tumor cells cohabit and codevelop with the molecular and cellular components of the surrounding microenvironment. In this plastic environment, the imbalance between pro-angiogenic and anti-angiogenic signaling guides the “angiogenic switch,” causing continuous generation of new blood vessels (173). The constant expression of pro-angiogenic factors does not allow blood vessel maturation resulting in the characteristic aberrant, leaky, disorganized, immature, thin-walled, and ill-perfused tumor vasculature (174). These aspects of tumor blood vessel cause and sustain a hypoxic microenvironment, even in highly vascularized tumors, that supports tumor growth favoring all hallmarks of the tumor, including immunosurveillance escape, metastasis, and not respond to therapies.

In addition to the well-known growth factors and vascular genes involved, angiogenesis is also regulated by epigenetic states of genes, and miRNAs expression and function (175). miRNAs act controlling the expression of angiogenesis-related factors, endothelial cell proliferation, migration, and tube formation. They are called angiomiRNAs (angiomiRs).

**Figure 1:**
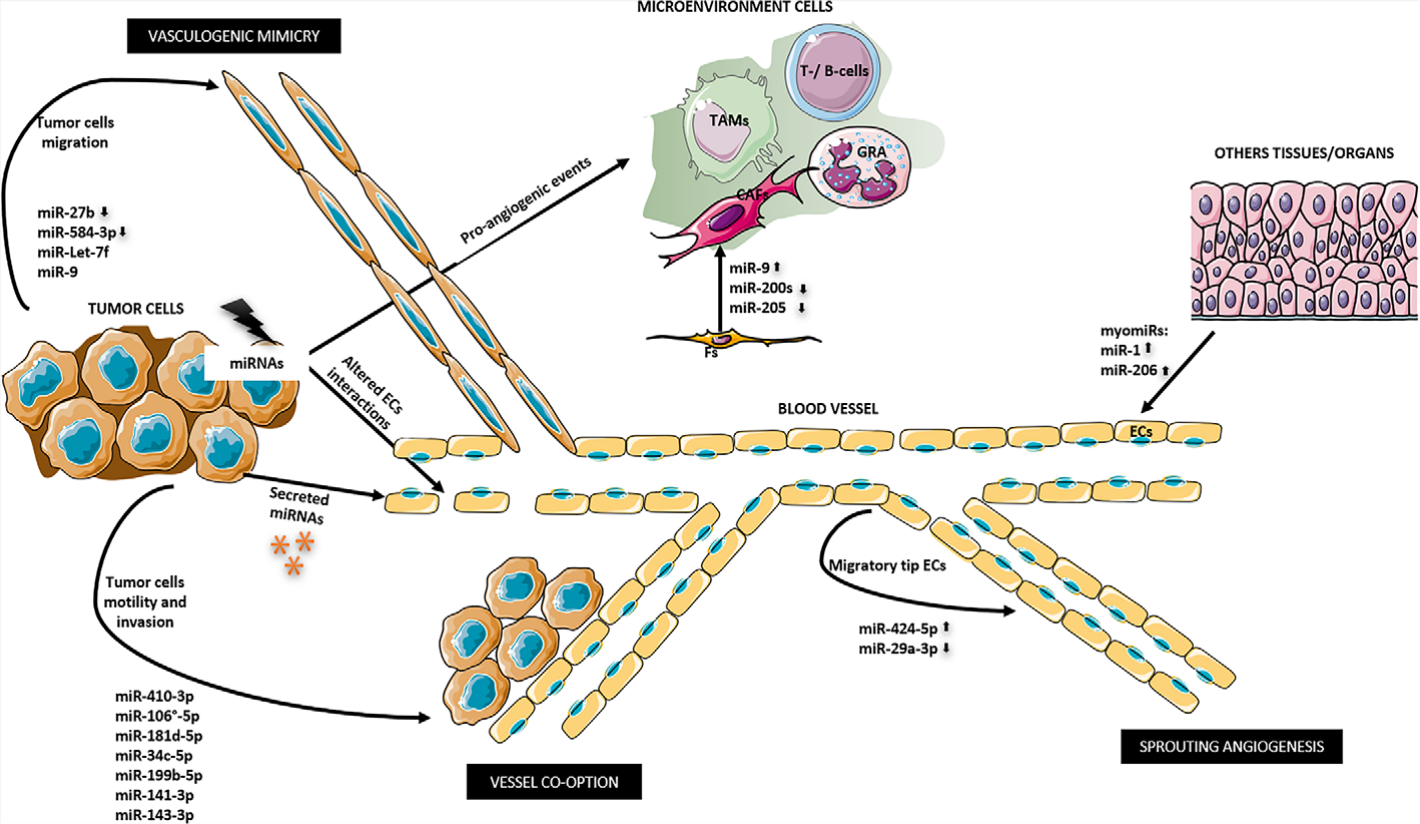
miRNAs ways to regulate tumor angiogenesis. 1) miRNAs derived from tumor cells indirectly may affect ECs supporting a pro-angiogenic microenvironment and altering ECs junctions. miR-9, miR-200s, and miR-205 promote the differentiation of fibroblasts into cancer associated fibroblasts (CAFs). 2) miRNA derived from ECs in autocrine manner may promote a migratory tip ECs phenotype that in turn promote sprouting angiogenesis. 3) Tumor-derived miRNAs may be secreted outside the cell and may directly regulate ECs. 4) Secreted miRNAs from other organs/tissues might affect ECs as myomiRs done. 5) Altered tumor-miRNA expression may promote tumor cell motility and invasion of the surrounding tissue that promote angiogenesis and tumor dissemination by vessel co-option. 6) Other miRNAs can induce vasculogenic mimicry. CAFs, cancer associated fibroblasts; ECs, endothelial cells; Fs, fibroblasts; GRA, granulocytes; TAMs, tumor associated macrophages; T-/B-cells.

It is possible to distinguish four-way of miRNAs action on tumor angiogenesis: i) tumor cells derived-miRNAs may affect the activity of ECs *via* non-cell-autonomous mechanisms; ii) ECs derived-miRNAs may regulate the cell-autonomous behavior; iii) tumor-derived extracellular vesicles transfer miRNAs to ECs; other organs/systems derived-miRNAs might affect either tumors or ECs (**Figure 1**). (176). In other words, in the first way, genetic and epigenetic alterations in tumor cells affect the phenotypes of ECs through changing microenvironment or intercellular interaction. In the second way, genetic and epigenetic alterations in ECs affect the cell’s own phenotypes. In the third way, tumor cells derived-miRNAs regulate the expression of pro- or anti-angiogenic factors in a paracrine manner by extracellular vesicles (EVs) that contain tumor cells-miRNAs which will be transferred from tumor cells to ECs, where they induce the pro-or anti-angiogenic effects. The fourth ways take advantage of cellular communication across tissues. miRNAs from other organs/systems, different from tumor cells or ECs, regulate the expression of pro- or anti-angiogenic factors in a paracrine manner. In zebrafish muscle, the evolutionarily conserved myomiRs, miR-1, and miR-206 from muscle cells negatively regulate angiogenesis by directly modulating VEGFA mRNA expression in ECs by a possible binding to three sites in its 3′UTR (177). Increased expression of miR-1 and miR-206 was found in patients affected by rhabdomyosarcomas and gastric cancer compared with healthy subjects (178, 179).

AngiomiRs can work targeting the negative regulators of angiogenesis and so promoting neoangiogenesis (pro-angiomiRs) or by targeting positive regulators of angiogenesis and so inhibiting it (anti-angiomiRs).

Besides the altered expression levels of certain angiomiRs, it has been shown that tumor angiogenesis can depend on the dysregulation of miRNAs biogenesis enzymes, Dicer and Drosha, involved in the maturation of ECs (180–182).

Recently Tiwari et al. (168) and Leone et al. (183) reviewed the mechanisms by which miRNAs regulate new blood vessel formation involving classical and non-classical growth factors and signaling pathways of angiogenesis. Therefore, we propose a concise table (**Table 2**) to get a glance at the main miRNA targets and tumors involved, and below, we examine the role of miRNAs in the three most studied mechanisms by which angiogenesis can happen, sprouting angiogenesis, vessel co-option and vasculogenic mimicry.

**Table 2:** AngiomiRNAs in tumors.

miRNA	Target	Function	Pro/anti-angiogenic
**Let-7b**	****Cdc34 EZH2**** ****ER-α36 HMGA1**** SOCS4	Overexpression modulates macrophage polarization and enhances tumor-associated macrophages to promote angiogenesis and mobility in *prostate cancer* (184)**.** Decreased expression correlates to high MVD and worse progression-free survival and overall survival in *NSCLC* (185).	pro/anti
**Let-7f**	TSP-1****	Overexpression attenuated the inhibition of angiogenesis by low-dose metronomic Paclitaxel chemotherapy in *breast cancer* (186)**.**	pro
**miR-9**	COL18A1**** MYC**** OCT4**** ****PTCH1 PHD3**** TSP2****	Overexpression in tumor cells and secretion by exosomes enhances proliferation and angiogenesis in *glioma* (187)**.**	pro
**miR-9-5p**	SOCS5****	Overexpression promotes angiogenesis and radiosensitivity in patients with *cervical cancer* (188 **).**	pro
**miR-10b**	HOXD10 NOTCH1 PAX6 TP53	****Overexpression retained independent prognostic significance for distant metastasis along with microvascular density (MVD) and vascular invasion in patients with *axillary lymph node−negative (ANN) breast cancer* (189)**.** Overexpression positively correlates to invasiveness, angiogenicity, and growth of the mesenchymal subtype-like glioma cells in *glioblastoma multiforme* (GBM) (190)**.**	pro
**miR-15a/16**	VEGFA	****Overexpression affects angiogenesis in *Multiple Myeloma* (191)**.**	anti
**miR-17-92**	HIF1α TGFBR2 VEGFA	Overexpressed in *colorectal cancer* in which inhibits tumor progression *via* suppressing tumor angiogenesis (192)**.**	anti
**miR-24**	Bim VEGF TGF−β AKT signaling β−catenin signaling	Overexpression promotes tumor growth and angiogenesis in *pancreatic cancer* (193)**.** Overexpression in conditioned medium from the U251 *glioma* cell line exhibited significantly increased cell viability, cell migration, and tube formation of HUVECs (194).	pro
**miR-126**	ADM Ang1 EGFL7 IGFBP2 MERTK VEGFA	Reduced expression correlates with increased angiogenesis and worse prognosis in *NSCLC* (185)*, breast cancer* (195), *gastric cancer* (196), and *cervical cancer* (197). Overexpression impairs vessel sprouting in h*epatocellular carcinoma* (198, 199). decreases angiogenesis and lymphangiogenesis in *oral cancers* (200), and is associated with beneficial effects in *cholangiocarcinoma* (201).	pro/anti
**miR-132**	CCND1 HB-EGF p21	Its expression inversely correlates to mast cell and microvessel density in *salivary gland tumors* (202). In a cluster with miR-212 inhibits tumor tissue proliferation and angiogenesis (203). Loss or reduced expression predicts poor overall and disease-free survival in patients with *primary osteosarcoma* (204).	anti
**miR-145**	N-RAS VEGF-A	Reduced expression positively correlates with malignancy stages of *breast tumors* (205) and tumor angiogenesis and progression in *uveal melanoma* (206).	anti
**miR-192**	EGR1 HOXB9	Overexpression induces inhibition of tumor angiogenesis in *multiple ovarian* and *renal tumor* models, resulting in tumor regression and growth inhibition (207).	anti
**miR-195**	IRS1 PRR11 PSAT1 VEGF	Overexpression inhibits tumor growth and angiogenesis in *breast cancer* (208), *Squamous Cell Lung Cancer* (SQCLC) cells (209), *prostate cancer* (210), and *colorectal cancer* (211). In *ovarian cancer*, it also reduces the cisplatin resistance (212).	anti
**miR-210**	EFNA3**** HIF-1α**** JAK2/STAT3 Pathway**** VHL SMAD4 STAT6 TET2	****Overexpression increases angiogenesis and promotes fibroblast transformation into cancer-associated fibroblasts (CAFs) in *lung cancer* (213)**.** Overexpression correlates with higher microvessel density in *HCC* (214), *malignant peripheral nerve sheath tumor* (MPNST) (215), and in *oral squamous cell carcinoma* (OSCC). In OSCC, its expression positively correlates with Leptin-receptor and HIF1- α expression (216). On the contrary, its overexpression down-regulates E2F3, and so inhibit tumor initiation in ovarian cancer cells (217).	pro
**miR-221**	ANG CXCL16 TSP2	Overexpression in cancer-derived exosomes promotes angiogenesis in *cervical squamous cell carcinoma* (218). Overexpression in *hepatocellular carcinoma* promotes angiogenesis (219).	pro
**miR-320**	Neuropilin 1 (NRP1)	Reduced expression inversely correlates with vascularity in *OSCC* (220) and *cholangiocarcinoma* (221).****	anti
**miR-296**	HGS PDGFRβ VEGFR2	Overexpression in *glioma* endothelial cells induces angiogenesis (222).****	pro
**miR-378**	MMP2 MMP9 Sufu VEGF	Overexpression is associated with cell migration, invasion, and tumor angiogenesis in patients affected by *NSCLC* with brain metastasis (223)****, in *ovarian cancer* (224 ****) and *glioblastoma* cell lines (225).	pro

Target’s abbreviations: ANG, Angiogenin; Ang1, Angiopoietin 1; ADM, Adrenomedullin; AKT, AKT Serine/Threonine Kinase; Bim, BCL2-like 11 apoptosis facilitator; CCND1, Cyclin D1; Cdc34, Cell Division Cycle 34; COL18A1, Collagen type XVIII alpha 1 chain; CXCL16, Chemokine C-X-C motif ligand 16; EFNA3, Ephrin-A3; EGFL7, Epidermal Growth Factor like domain 7; EGR1, Early Growth Response 1; EZH2, Enhancer of zeste homolog 2; E2F3, E2F Transcription Factor 3; ER-α36, estrogen receptor-α36; Fus1, nuclear fusion protein; HB-EGF, Heparin-Binding EGF-Like Growth Factor; HGS, Hepatocyte growth factor-regulated tyrosine kinase substrate; HIF-1α, Hypoxia-inducible factor 1-alpha; HMGA1, High Mobility Group AT-Hook 1; HOXB9 Homeobox B9; HOXD10, Homeobox D10; IGFBP2, Insulin-Like Growth Factor Binding Protein 2; IRS1,Insulin receptor substrate 1; JAK2/STAT3, Janus Kinase 2/Signal transducer and activator of transcription 3; MERTK, MER proto-oncogene, tyrosine kinase; MYC, Myc proto-oncogene protein; MMP2/-9, metalloproteinases; N-RAS, Proto-Oncogene, GTPase; NRP1, Neuropilin 1; PAX6, Paired Box 6; PHD3, Prolyl-hydroxylase 3; PRR11, Proline rich 11; PSAT1, Phosphoserine aminotransferase 1; PTCH1, Patched 1; OCT4, octamer-binding transcription factor 4; SMAD4, SMAD Family Member 4; SOCS-4/-5, Suppressor of cytokine signaling -4/-5; STAT6, Signal transducer and activator of transcription 6; Sufu, suppressor of fused; TET2, Tet Methylcytosine Dioxygenase 2; TGF-β, transforming growth factor β; TGFBR2, Transforming Growth Factor, Beta Receptor II; TP53, Tumor Protein P53; TSP-1/-2, Thrombospondin-1/-2; VEGF/-A, Vascular Endothelial Growth Factor/-A; VEGFR2, Vascular endothelial growth actor receptor 2; VHL, Von Hippel-Lindau.

Sprouting angiogenesis requires detailed temporal coordination of ECs migration and proliferation (171). In this mechanism, new blood vessels originate from the pre-existing one, and the two main actors are tip cells and stalk cells. The tip cells are activated ECs on the vascular front that migrate in response to angiogenic stimuli coming from the surrounding microenvironment. The stalk cells are activated ECs which are located behind the vascularization front, intensely proliferate and organize themselves to form the vessel lumen. Quiescent ECs became tip/stalk cells in response to vascular endothelial growth factor-A (VEGF-A) gradient concentration and its receptor (VEGFR) expression level in association with extensively transcriptomic alterations (226). *In vitro*, by a 3D-spheroid model that reproduces the initial phase of sprouting angiogenesis and by the administration of VEGF-A, it was demonstrated that quiescent ECs are activated in response to VEGF-A and differentiate into tip cells that allow the migratory process rather than cell proliferation (227). In this phenotypic switch, during initial sprouting angiogenesis, miRNA plays a pivotal role in controlling tip cell fate by activating specific gene programs (227). The computational method Gene Set Enrichment Analysis (GSEA) showed the activation of genes that regulate extracellular matrix organization, cell adhesion molecules, integrin pathway, protein translation, and collagen formation. Instead, the genes that are associated with cell-cycle progression or are involved in mitogen-associated protein kinase (MAPK) signaling are repressed (227, 228). miR-424-5p up-expression and miR-29a-3p down expression were reported as the primary miRNAs in this network where VEGF triggers tip cell specification (227). miR-424-5p expression induces the repression of target genes involved in cell proliferation, including MAPK and cell cycle-related ones (227, 229). On the contrary miR-29a-3p downregulation induces the expression of target genes involved in cell migration as extracellular matrix (ECM) -related ones (227, 230). The same authors investigated the angiogenic post-transcriptional activity of 58 sprouting-associated genes (including the two mentioned above) in human colorectal cancer, by the means of GSEA, showing the utility of miRNA network analysis for patients therapy stratification and new druggable biomarkers detection.

Vessel co-option is an alternative mechanism of angiogenesis in which cancer cells migrate towards and along the pre-existing vasculature without or before angiogenesis, or in response to anti-angiogenic therapy (231). In addition to promoting tumor growth and progression, vessel co-option mediates tumor resistance to anti-angiogenic therapy (232). In a study, in which was evaluated the role of vessel co-option concerning sorafenib [a tyrosine kinase inhibitor (TKI)] resistance in hepatocellular carcinoma, by miRNA sequencing and qRT-PCR, four signaling pathways that are up-regulated were found (233). These pathways, involved in cellular motility and invasion, were including axonal guidance, EMT, STAT3, and Wnt/βcatenin signaling. Evaluating the miRNA cluster involved in EMT, three transcripts coding for Vimentin, and Zinc Finger E-Box Binding Homeobox-1/-2 (ZEB1 and ZEB2), were found significantly up-regulated in resistant tumor vs. control (233). These results highlight the importance of targeting different types of angiogenesis mechanisms because the tumor can activate alternative ways that non respond to classic anti-VEGF or TKI therapies.

Vasculogenic mimicry is a tumor neovascularization mechanism different from the endothelium-dependent ones. This mechanism is often present in highly aggressive and genetically dysregulated tumors in which tumor cells mimic endothelial cells. In ovarian cancer were found that low miR-27b expression levels are closely correlated with high vascular endothelial cadherin (VE-cadherin) expression and the high vasculogenic mimicry capability of tumor cells (234). VE-cadherin has been recognized as a master gene of tumor vasculogenic mimicry (235, 236). MiR-27b targets the 3′ UTR of VE-cadherin mRNA to suppress ovarian cancer cell migration and invasion as well as vasculogenic mimicry, as also demonstrated in hepatocellular carcinoma (234, 237).

Another miRNA related to vasculogenic mimicry is miR-584-3p. This miRNA function as a potent tumor suppressor by inhibiting vasculogenic mimicry of malignant glioma targeting Rho Associated Coiled-Coil Containing Protein Kinase 1(ROCK1) that disturb hypoxia-induced stress fiber formation and migration of glioma cells (238). In glioma, vasculogenic mimicry could also be suppressed by miR-Let-7f disturbing periostin-induced migration of tumor cells and by miR-9 disturbing Stathmin (a cytosolic phosphoprotein which regulates microtubule dynamics during cell-cycle progression) expression in vascular endothelial cells and glioma cells (239–241).

No specific studies were found concerning miRNA and tumor angiogenesis that occurs by intussusception or vasculogenesis.

In addition to the proliferation and migration of ECs and tumor cells to promote angiogenesis, miRNAs can control new blood vessel formation by regulating the tumor microenvironment. This means that miRNAs can regulate all the cellular and non-cellular components of the surrounding tissue. A recent review of Pan et al. elucidates the role of miRNA in regulating cancer-associated fibroblasts (CAFs), tumor-associated bone marrow mesenchymal cells, tumor-associated macrophages (TAMs), T-cells, and ECs (242). For instance, in breast cancer, it was shown that several miRNAs are implicated in the switch from normal fibroblast to CAFs, including miRNA-9 (up-regulated), miR-200s, and miR-205 (down-regulated). The reduced expression of miR-205 converts the normal fibroblasts into CAFs by promoting YAP1 expression, which is also involved in angiogenesis (243).

Summing up, miRNAs provide new insights into understanding the molecular mechanisms concerning angiogenesis. Their dysregulation may be responsible for the failure of current anti-angiogenic therapy.

In addition to targeting the different types of angiogenesis mechanisms, the new therapeutic approach should also take into account the vascular morphology. As said above, the tumor blood vessels are not mature, so vascular normalization is necessary. Vascular normalization can be accomplished by attenuation of hyperpermeability, increasing vascular pericyte coverage, inducing a more normal basement membrane, resulting in improved tumor perfusion, reduced hypoxia, and therapeutic outcomes (244).

Therapeutic strategies involve the use of miRNA as antagonists or mimics. Antagonists act inhibiting endogenous miRNAs with a gain-of-function in disease. Mimics act simulating miRNAs with a beneficial effect, but that are lost in the disease. miRNAs antagonists or mimics may be employed in transient normalization of tumor vasculature to make it more competent for oxygen and drug delivery (245).

### miRNA in Lymphoma

Lymphoma is a group of tumors that begins in the cells of the immune system, lymphocytes-T and -B, that grow out of control. These cells reside in the lymph nodes, spleen, thymus, bone marrow, and other parts of the body. All these parts of the lymphatic system can be affected by lymphoma.

There are two main types of lymphoma, Non-Hodgkin Lymphoma (NHL) and Hodgkin Lymphoma (HL) (246, 247). The main discriminant between the two types of lymphoma is the presence of a specific type of abnormal cells called Reed-Sternberg cells. These cells are also known as lacunar histiocytes and are giant cells found by light microscopy in biopsies from individuals affected by HL. In NHL, these cells are not present. Moreover, other features distinguish the two major groups of lymphoma are: NHL is more common than Hodgkin lymphoma; the median age for diagnosis of NHL is 55 whereas for HL is 39; NHL may start in lymph nodes anywhere in the body, whereas HL usually starts in the upper part of the body (neck, chest or armpits); NHL is typically not diagnosed before it has reached a more advanced stage, whereas, HL is often diagnosed at an early stage and consequently it is one of the most treatable cancers.

Despite these differences, both NHL and HL have similar symptoms, like enlarged lymph nodes, fatigue, weight loss, and fever. In both, different subtypes are distinguished (246, 247). NHL could affect adults, children, and non-lymphatic tissue as skin. Primarily, they are classified as B- or T-cell lymphoma based on the white blood cell category affected, and secondarily as indolent or aggressive based on how tumor cells grow and spread (slow or quickly, respectively) (248). HL subtypes are Nodular sclerosis Hodgkin lymphoma (NSCHL), Mixed cellularity Hodgkin lymphoma (MCCHL), Lymphocyte-rich Hodgkin lymphoma, and Lymphocyte-depleted Hodgkin lymphoma (248).

Lymphocytes, like all the other classes of blood cells, are derived from bone marrow resident hematopoietic stem cells (HSCs), through progressive loss of pluripotency caused by a response to lineage determining signals. Intrinsic and extrinsic molecules, including miRNAs, regulate cell fate and lineage commitment in stem and progenitor cells (249). miRNAs dysregulation has been found in both solid and hematologic tumors by various genome-wide techniques, including oligonucleotide miRNA microarray analysis, bead-based flow-cytometric technique, quantitative real-time PCR for precursor/active miRNAs, miRAGE or RAKE assay (250–253). Metzler et al. provided the first evidence of miRNA involvement in human lymphoma. In children with Burkitt lymphoma, He and Coworkers found that miR‐155 was 100-fold increased compared to healthy blood and pediatric leukemia patients (254).

Different studies have demonstrated the essential role of Dicer1 for stem cell persistence and differentiation *in vivo*. In HSCs, Dicer1 deletion compromises the hematopoietic stem/progenitor cell (HSPC) function in a manner consistent with stem cell death (255). In the same work, a specific miRNA, miR-125a, was found to control the HSCs population size by regulating HSPCs apoptosis. In fact, miR-125a protects HSPCs from apoptosis and promotes the extensive expansion of the HSCs (255). Moreover, the ablation of Dicer1 in B- cell progenitors blocks the transition from pro- to pre-B cells because of mir-17~92 targeting the pro-apoptotic molecule Bim (256). Other miRNAs of the molecular circuitry that controls hematopoiesis are miR-223, miR-181, and miR-142. These miRNAs are specifically expressed in hematopoietic cells, and their expression is dynamically regulated during early hematopoiesis and lineage commitment (257). In detail, the Authors have shown that miR-181 is preferentially expressed in the B-lymphocytes of bone marrow, and its expression in HSPCs led to an increased fraction of B-lineage.

miRNAs are also profiled in several distinct stages of T-lymphocyte development. miR-181a represses, biding their 3′ UTR elements, the expression of Bcl-2, CD69, and the T-cell receptor genes, all of which are coordinately involved in positive selection of CD4^+^/CD8^+^ cells during thymocyte maturation (258, 259).

miR-155 regulates both B- and T-lymphocytes functions. Its deletion causes immunodeficiency because of B-lymphocytes produce lower levels of immunoglobulins, tumor necrosis factor (TNF) and lymphotoxin, and because of T-lymphocytes produce lower levels of interferon-gamma (IFNγ) and interleukin-12 (IL-12) promoting differentiation to Th2 phenotype (260, 261). For miR-155 is currently underway the first-in-human phase 1 clinical trial (Identifier NCT02580552) and phase 2 clinical trial (Identifier NCT03713320). The biopharmaceutical company, MiRagen Therapeutics is developing a locked nucleic acid-modified oligonucleotide inhibitor of miR-155, called MRG-106, and also known as Cobomarsen. The preliminary results have shown that miR-155, which is usually repressed in lymphoma, might be de-repressed by Cobomarsen in patients with mycosis fungoides, the most common form of cutaneous T-cell lymphoma. miR-155 overexpression will deactivate/activates signaling pathways, including JAK/STAT, MAPK/ERK, and PI3K/AKT, associated with tumor cell proliferation, survival, and apoptosis (262).

Angiogenesis in hematological malignancies, as in solid tumors, correlates with tumor growth and development (263). As discussed in the previous section, miRNAs regulate various aspects of angiogenesis, including proliferation, migration, and morphogenesis of endothelial cells. Therefore, the knowledge of all these mechanisms will permit to improve the management of malignancies, and therefore the patients’ prognosis by targeting specific molecules and processes involved in tumor angiogenesis. Despite the several data on miRNome characterization in lymphoma, the data about the specific regulation of angiogenesis by miRNAs are scarce (264–266).

miR-135b up-regulation was demonstrated in anaplastic large-cell lymphoma (ALCL) cell lines and clinical samples positive to the oncogenic translocation nucleophosmin-anaplastic lymphoma kinase (NPM-ALK) (267). NPM-ALK promotes the expression of miR-135b and its host gene LEMD1 through STAT3 activation. By the decoy RNA system to achieve long-term miR-135b suppression and by GSEA, it was shown that miR-135b targets FOXO1, TGFBR1, SIRT1, cyclin G2, CREG1, Bcl11b, and STAT6 and confer chemoresistance to ALCL cells. Moreover, NPM-ALK/STAT3–miR-135b axis polarizes the identity of ALCL cells to a Th17 immunophenotype (pro-inflammatory cells) downregulating GATA3 and STAT6. This pro-inflammatory immunophenotype shift was also demonstrated in a xenograft model of ALCL in which the tumor growth and angiogenesis support and are supported by the pro-inflammatory microenvironment (267, 268).

In the same tumors, ALK-positive ALCL, the angiogenesis is still sustained by hypoxia-miR-16 downregulation. miR-16 downregulates VEGF expression biding the 3’UTR- of its mRNA directly. In onco-ALK mouse MEF cell lines and transgenic models, it was shown that ALK and hypoxia-inducible factor 1 alfa (HIF-1α) co-expression induce miR-16 downregulation, which therefore does not allow VEGF downregulation by post-transcriptional regulation (269).

The polycistronic miR-17-92 cluster, encoding six miRNAs (miR-17, miR-18a, miR-19a, miR-19b, miR-20a, and miR-92), is involved in immune cell development, have an oncogenic role in lymphoma, and a controversial role in angiogenesis (270, 271). In p53-null colonocytes, co-expressing K-RAS and c-Myc, was shown an increased neoangiogenesis correlated with the downregulation of anti-angiogenic thrombospondin-1 (Tsp1) *via* miR-18, and of the connective tissue growth factor *via* miR-18 and miR-19 (272). On the contrary, other authors demonstrated an anti-angiogenic role of the same cluster showing that the overexpression of miR-20a inhibits sprouting *in vitro*, whereas its inhibition increases ECs sprout formation in perfused vessels Matrigel plugs, but not following systemic inhibition (273). The dispute can be overcome, considering that tumor angiogenesis regulation is context-dependent.

**Figure 2:**
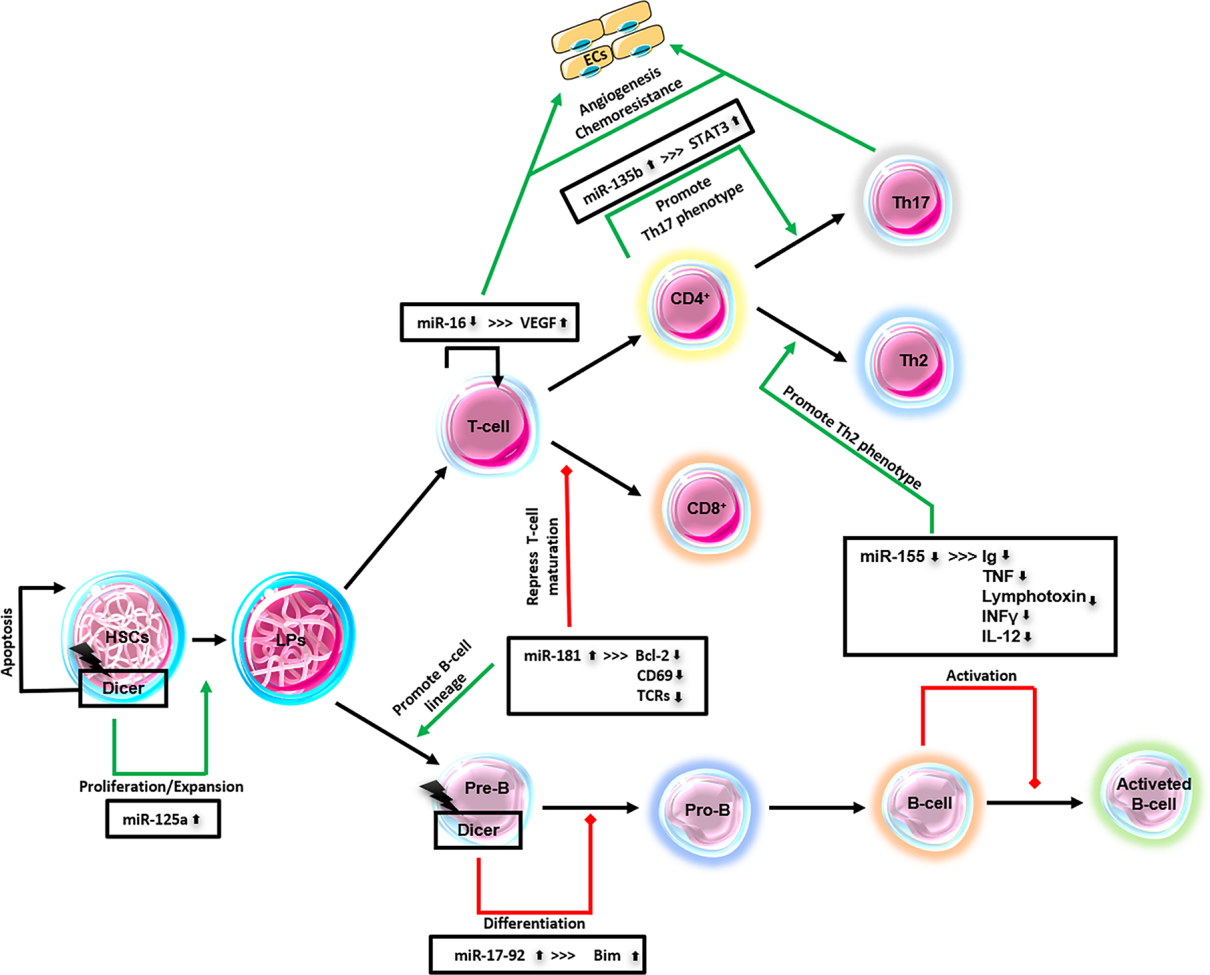
Major lymphoma-derived miRNAs. This schematic diagram represents miRNAs that have been identified in lymphomas and their relationship to lymphopoiesis. ECs, endothelial cells; HSCs, hematopoietic stem cells; LPs, lymphocytes precursors; Pre-B, pre-B-cells; Pro-B, pro-B-cells; CD4+, T-cells; CD8+, T-cells; Th2, T-helper cells; Th17, T-helper cells; T-/B-cells.

A schematic diagram of the significant lymphoma-miRNAs above mentioned is represented in **Figure 2**.

## Challenges and Future Directions

Large-scale gene expression analysis evidence that the expression of individual miRNA or miRNA signatures will become routine biomarkers for cancer diagnosis and prognosis, and personalized therapy, thanks to their accessibility, high specificity, and sensitivity.

miRNAs induce epigenetic alterations that are reversible and targeted multiple regulators of related pathways (274–277). Tumor angiogenesis can be ruled by derepressing/miming tumor suppressor genes with angiostatic properties that are instead epigenetically silenced in a tumor, endothelial, and/or microenvironment cells, or by repressing oncogenes codifying for pro-angiogenic molecules (278, 279). Some argue that miRNA inhibitors are prevalently used for therapies because endogenous miRNA inhibition is less hazardous than overexpressing. On the contrary, others sustained that miRNA therapy to replace endogenous miRNAs repressed in cancer cells, using miRNA mimics, is less dangerous because they will not damage normal cells since they already express it.

miRNAs for therapy are usually chemically modified to stabilize the oligonucleotide once injected by intravenous, intraperitoneal, or intramuscular ways. miRNAs could be embedded in a liposome or viral backbone, or associated with targeted nanoparticles to improve affected cells’ yield.

Anti-angiogenic therapies usually directly targeted ECs, because ECs are in direct contact with blood carrying drugs, a small amount of ECs regulate many tumor cells, ECs are genetically more stable than tumor cells and so therapeutic effects are more predictable, and finally, only the ECs in the tumor site are activated and could be specifically targeted. Tumor ECs “angiogenic stage-related profiles” was extensively characterized by *in vitro* ECs culture under different cytokines and growth factors stimuli, using tube formation models, and ECs isolated from *in vivo* sources (280–282).

miRNA combination therapies with anti-angiogenic strategies, chemotherapy, radiotherapy, photodynamic therapy, and immunotherapy are in-depth reviewed elsewhere (278, 283). Noteworthy are two approved clinical trials about miR-16 and miR-34a. miR-16-based microRNA mimic is employed in a clinical trial to treat malignant pleural mesothelioma and non–small-cell lung cancer (Identifier NCT02369198). miR-34a mimic, in combination with the antiangiogenic drug doxorubicin, is in phase I clinical trial to treat patients with advanced solid and hematological tumors (Identifier NCT01829971).

Given the scarcity of miRNAs in clinical studies, their clinical application is not easy. The significant challenges are to prevent unwanted off-target effects, to develop an efficient and specific miRNA delivery system, to optimize effective dose (167). Well-defined pre-clinical studies, using cell lines and animal models and a broad set of human samples, are needed to solve these challenges. For example, coculture and 3D-culture for ECs with other microenvironment cells to study angiogenesis represents a more physiological condition. In order to improve clinical trials, it will be necessary to follow miRNA-treated patients for a long time to prove that miRNAs are unique biomarkers of angiogenesis‐related cancers. Moreover, miRNAs signatures from serum, plasma, or urinary should be further investigated as less invasive procedures. Recently, circulating exosomal miRNAs, thanks to reasonable stability, higher specificity, and sensitivity, appear potential biomarkers for clinical applications.

Standardized miRNAs application protocols in clinical practice are missing, maybe due to the lack of extensive studies on miRNAs with limited data comparability. More attention must be paid to selecting the starting material, sample collection, detection platform, and statistical analysis (284). For instance, if we wanted to quantify the circulating miRNAs, we should be attentive to the source that could be the whole blood, serum, plasma, exosomes, or microvesicles. When whole blood is used, miRNAs from blood cells will influence the total amount of the only circulating ones (273, 274). For sample collection, extreme pH changes or repeated freezing and thawing cycles should be avoided. The best commercial kit available for miRNAs extraction should choose to produce the highest yield of total RNA extraction (275). Moreover, it must be considered that in the samples of cancer patients, there will be a greater quantity of miRNA, and therefore to compare the miRNA expression data with healthy ones, it is more appropriate to use the same volume of starting material (biological fluid, cells, tissue) instead to use the same amount of total RNA.

Normalization of miRNAs quantification is another challenge. The small nuclear U6, or small nucleolar RNAs SNORD44, or some validated miRNAs, as miR-16, were usually used for normalization, but it was demonstrated that these common reference genes for miRNA research are poor normalizers because differentially regulated (285, 286). Therefore, it is necessary to verify the expression stability of putative normalizers in each experiment.

Apart from these technical problems, the difficulties in translate microRNA biomarkers from bench to clinic are associated with the inconsistency of miRNAs as unique biomarkers since: several of them are detectable in patients with different tumor types; other studies have shown different results for the same miRNA in the same tumors; sometimes is difficult to discriminate among closely related miRNAs (286, 287).

In order to improve and implement the clinical application of miRNAs, it is necessary to start with a careful evaluation of the preliminary study approaches. miRNAs that are already known and that are present in the miRNA database (miRbase) can be detected and quantified by polymerase chain reaction (PCR)-based or hybridization-based methods. Quantitative reverse transcription-polymerase chain reaction, qRT-PCR, is the gold-standard method in miRNA profiling in human (288). TaqMan TLDA microfluidic cards and miRCURY LNA qPCR detection platforms are the most used (276). Hybridization techniques are also used. miRNA microarrays analyze thousands of miRNAs in one assay but have less sensitivity than PCR-based methods (289).

On the other hand, to know and quantify new miRNAs, miRNA-seq is applied (290). The computational and prediction methods developed to detect tumor-related miRNAs are not satisfactory enough (291). However, despite the improvement in miRNA expression profiling technologies and big data in public databases, to date, these public portals with miRNA expression data from patients around the world are underemployed.

## Conclusions

The human genome encodes approximately 2,600 mature miRNAs and more than 200,000 mRNAs (279). miRNAs may target more than one mRNAs, thus influencing multiple molecular pathways, but also mRNAs may bind to a variety of miRNAs, either simultaneously or in a context-dependent manner (292, 293).

Computational analysis suggests that around 30% of protein-coding genes in humans are regulated by miRNAs (177). Each step of miRNA biogenesis, including miRNA transcription, processing by Drosha and Dicer, transportation, RISC binding, and miRNA decay, is finely controlled. Therefore, minimal dysregulation in miRNA biogenesis and their control of gene expression can lead to tumorigenesis.

Here we have provided updates in miRNAs biogenesis, function, and role in health and tumor and especially in angiogenesis. The identification of miRNAs as onco-miRNAs or oncosuppressor-miRNA with a critical role in angiogenesis has raised new hopes for the treatment of tumors. They may be candidates even for target-specific therapies as antagonists or mimics (294). However, their diffusion as drugs is still far from the full clinical application because there are several criticalities to be carefully considered as tissue-specific delivery and cellular uptake sufficient to produce the therapeutic effect.

The relationship between angiogenesis and progression of several hematological malignancies is well established, and anti-angiogenic drugs appear a promising therapeutic strategy (295). Endostatin, immunomodulatory drugs (e.g., Thalidomide), neutralizing antibodies (e.g., Bevacizumab), and histone deacetylase inhibitors (e.g., Vorinostatat) are used in the treatment of lymphoma, but they are not enough to re-establish “normal” angiogenesis. miRNAs could cover part of the leaks. Among the hematological tumors, we decide to evaluate the literature concerning the role of miRNAs in the regulation of angiogenesis in lymphoma, and we observed that although the studies are scarce, there are two clinical trials for miR-155 in progress.

In addition to the critical issues concerning the pharmacokinetics and pharmacodynamics of miRNA-drugs, it should be stressed that there is still much to discover in the field of miRNAs. The sequencing of miRNome and the mapping of miRNA–mRNA interactions is far from being complete due to the recognized challenge of computational prediction of mRNA–microRNA interactions. In most studies, methods of high-throughput analysis based on Next Generation Sequencing technology (NGS) and computational analysis are applied. However, these experimental approaches to define the role of small non-coding RNAs in the biological and pathological processes are not enough. The morphology-based approach, such as dual *in situ* Dual ISH-IHC Technology, should walk together with the most modern computational and molecular technologies for a more precise characterization of the biological events in a more realistic molecular context (296–298).

## Author Contributions

This work was conceived and planned by TA and DR. The original draft preparation and writing were made by TA, RT, and MG. DR reviewed and edited the manuscript. All authors contributed to the article and approved the submitted version.  

## Conflict of Interest

The authors declare that the research was conducted in the absence of any commercial or financial relationships that could be construed as a potential conflict of interest.

## Abbreviations

AML, acute myeloid leukemia; Ago, argonaute family of proteins; AEG-1, astrocyte elevated gene-1; ALCL, anaplastic large-cell lymphoma; AML, acute myeloid leukemia; ANN, axillary lymph node−negative; B-CLC, B-cell chronic lymphatic leukemia; CAFs, cancer-associated fibroblasts; CSCs, cancer stem cells; DGCR8, DiGeorge syndrome critical region 8; DNMT, DNA methyltransferase; dsRNA, double-stranded RNA; ECM, extracellular matrix; EGCG, epigallocatechin-3-gallate; ECs, endothelial cells; EMT, epithelial-mesenchymal transition; ESCs, embryonic stem cells; FBXW7, F-box/WD repeat-containing protein 7; FMR1, fragile X mental retardation 1; GBM, glioblastoma multiforme; GEMIN4, gem nuclear organelle associated protein 4; GSEA, Gene Set Enrichment Analysis; HCC, hepatocellular carcinoma; HDAC, histone deacetylase; HIF-1α, hypoxia-inducible factor 1 alfa; HL, Hodgkin lymphoma; HSC, hematopoietic stem cells; HSPC, hematopoietic stem/progenitor cell; IFNγ, interferon-gamma; IL-12, interleukin-12; IPO8, importin 8; lncRNA, long non-coding RNA; MAPK, mitogen-activated protein kinase; MCCHL, mixed cellularity Hodgkin lymphoma; miRISC, miRNA-RISC; miRNA, microRNAs; MLL, myeloid/lymphoid leukemia or mixed-lineage leukemia; mRNA, messenger RNA; MVBs, multivesicular bodies; MVD, microvascular density; NES, nuclear export signal; NHL, non-Hodgkin lymphoma; NLS, nuclear localization signal; NPM-ALK, nucleophosmin-anaplastic lymphoma kinase; NSCHL, nodular sclerosis Hodgkin lymphoma; NSCLC, non-small cell lung cancer; nSMase2, neutral sphingomyelinase 2; OSCC, oral squamous cell carcinoma; pADPr, poly-ADP-ribose; PARG, poly-ADP-ribose glycohydrolase; PARylation, poly-ADP-ribosylation; PARPs, poly-ADP-ribose polymerases; PCDH9, Protocadherin 9; pre-miRNA, precursor miRNA; pri-miRNA, primary miRNA; pRNA, promoter-associated RNA; PTEN, phosphatase and tensin homolog; PTMs, post-translational modifications; Ran-GTPase, RAS-related nuclear protein-guanosine-5’-triphosphate-ase; RBPs, RNA- binding proteins; rER, rough endoplasmic reticulum; RISC, RNA-induced silencing complex; RNAi, RNA interference; ROCK1, Rho-associated coiled-coil containing protein kinase 1; R2D2, dsRNA binding protein with two dsRNA binding domains; SAHA, suberoylanilide hydroxamic acid; shRNA, short hairpin RNA; SND1, staphylococcal nuclease domain-containing protein 1; snoRNAs, small nucleolar RNAs; SNPs, single-nucleotide polymorphisms; SQCLC, squamous cell lung cancer; TAMs, tumor-associated macrophages; TGA, transcriptional gene activation; TGS, transcriptional gene silencing; TCGA, the cancer genome atlas; TKI, tyrosine kinase inhibitor; TMAs, tissue microarrays; TNF, tumor necrosis factor; TNRC6A, trinucleotide repeat-containing adaptor 6a; tRFs, tRNA derived RNA fragments; tRNAs, transfer RNAs; tsRNAs, tRNA-derived small RNAs; UTR, untranslated region; VE-cadherin, vascular endothelial cadherin; VEGF, vascular endothelial growth factor; VEGFR, vascular endothelial growth factor receptor; VIG, vasa intronic gene; XPO5, exportin5; ZEB1/2, zinc finger e-box binding homeobox 1/2.
